# Supplementary material for: Miga-mediated endoplasmic reticulum–mitochondria contact sites regulate neuronal homeostasis
Source: eLife. 2020 Jul 10;9:e56584. doi: 10.7554/eLife.56584 (PMC7556861; doi:10.7554/eLife.56584)
Supplement: Supplementary file 1. [file elife-56584-supp1.docx]

**Supplemental Materials**

**The genotypes of the fly strains used in this study:**

**Fig.1A, 1A’, 1B and 1B’ *CTL:*** *GMRGal4/UAS-Attp40;* **1C,1C’, 1D,1D’**

*GMRGal4/UAS-MigaRFP;* ***1E,1E’, 1F, 1F’*** *GMRGal4/UAS-MarfMyc;*

***1G,1G’, 1H, 1H’*** *GMRGal4/UAS-MitoPLD-HA;*

**Fig. 2E, 2K:** *CgGal4/UAS-FLAG.Vap33-1.HA;* **Fig. 2F, 2L, 6G, 6I:** *CgGal4/UAS-MigaRFP;*

**Fig. 2G, 2M:** *CgGal4 UAS-MigaRFP/UAS-FLAG.Vap33-1.HA;* **Fig. 2H, 2P:** *CgGal4/UAS-* *Miga^FM^RFP;* **Fig. 2I:** *CgGal4 UAS-Miga^FM^RFP/ UAS-FLAG.Vap33-1.HA;* **Fig. 2N:** *CgGal4/+;UAS-Vap33RNAi/+；***Fig. 2O:** *CgGal4 UASMigaRFP/+; UAS-Vap33RNAi/+* **; Fig.2J, 6F, 6H:** *CgGal4/UAS-attp40.*

**Fig.3A, 3A’, 3M, 3Q, 6M:** *GMRGal4/UAS-Attp40;* **Fig.3B, 3B’, 3N, 3R, 6N:** *GMRGal4/UAS-MigaRFP;* **Fig.3C, 3C’:** *GMRGal4/UAS-Miga^FM^RFP;* **Fig.3D, 3D’:** *GMRGal4/UAS-MigaRFP;Vap33RNAi/UAS-DCR2;* **Fig.3E, 3E’:** *GMRGal4/+;Vap33RNAi/UAS-DCR2;* **Fig.3H:** *CgGal4/UAS-attp40;* **Fig.3I:** *CgGal4/UAS-MigaRFP;* **Fig.3J:** *CgGal4/UAS-PTPIP51RFP;* **Fig.3K:** *CgGal4/UAS-TetherRFP;* **Fig.3O, 3S:** *GMRGal4/UAS-PTPIP51RFP;* **Fig.3P, 3T:** *GMRGal4/UAS- TetherRFP;*

**Fig.4A:** *Iso/Y;* **Fig.4B:** *Miga mu/Y;* **Fig.4F, 4J:***yw, isoFRT19A/ Cl(1) FRT19A; eyFlp/+;* **Fig.4G, 4K:** *yw, Miga mu FRT19A/Cl(1) FRT19A; eyFlp/+*

**Fig.4H, 4L:** *Miga mu FRT19A/Y; Miga-gRes-3HA*; **Fig.4I, 4M:** *Miga mu FRT19A/Y; Miga^FM^gRes-3HA.*

**Fig.6A:** *CgGal4/UAS-Miga8S-ARFP;*

**Fig.6O:** *GMRGal4/UAS-Miga I-III^SA^ RFP;* **Fig.6P:** *GMRGal4/UAS-Miga I-III^SE^RFP*

**Fig.7D, 7F:** *CgGal4 UAS-MitoGFP/ UAS-Attp40;* **Fig.7E, 7G:** *CgGal4 / UAS-MIGA2RFP.*

**Fig.S1A: *CTL:*** *Mef2Gal4/UAS-Attp40;* ***UAS-Miga-RFP:*** *Mef2Gal4/UAS-MigaRFP;* ***UAS-Miga^FM^-RFP:*** *Mef2Gal4/UAS-Miga^FM^RFP;*

**Fig.S2D: *CTL:*** *CgGal4/UAS-Attp40;* ***Miga-RFP:*** *CgGal4Gal4/UAS-MigaRFP;* ***MigaI-III^SA^-RFP:*** *CgGal4/UAS-MigaI-III^SA^RFP;* ***MigaI-III^SE^-RFP:*** *CgGal4/UAS-MigaI-III^SE^RFP;*

**Fig.S2H: *CTL:*** *Mef2Gal4/UAS-Attp40;* ***Miga-RFP:*** *Mef2Gal4/UAS-MigaRFP;* ***MigaI-III^SA^-RFP:*** *Mef2Gal4/UAS-MigaI-III^SA^RFP;* ***MigaI-III^SE^-RFP:*** *Mef2Gal4/UAS-MigaI-III^SE^RFP;*
